# Development and validation of a novel short-form nutrition literacy measurement tool for Chinese college students

**DOI:** 10.3389/fpubh.2022.962371

**Published:** 2022-09-09

**Authors:** Guangju Mo, Siyue Han, Tianjing Gao, Qing Sun, Min Zhang, Huaqing Liu

**Affiliations:** ^1^School of Public Health, Bengbu Medical College, Bengbu, China; ^2^The First Affiliated Hospital of Bengbu Medical College, Bengbu, China; ^3^School of Health Management, Bengbu Medical College, Bengbu, China

**Keywords:** nutrition literacy, NL-SF12, China, college students, measurement tool

## Abstract

**Objectives:**

To develop and validate a short-form nutrition literacy (NL) assessment tool for Chinese college students based on a 43-item NL measurement scale.

**Methods:**

To develop and validate short-form NL scale, 1359 college students were surveyed, the data were analyzed using exploratory factor analysis, linear regression analysis, Item analysis, confirmatory factor analysis, and Pearson correlation.

**Results:**

The 12-item short-form NL scale (NL-SF12) was developed using factor analysis and regression analysis, which accounted for 96.4% of the variance. The correlation coefficient between the NL-SF12 and NL-43 was 0.969, indicating satisfactory criterion-related validity. The NL-SF12 had a Cronbach's α of 0.890, suggesting strong internal consistency reliability, and content validity index was greater than 0.9, indicating that each domain accurately reflects the connotation of nutrition literacy. The model–data fit and convergent validity of the confirmatory factor analysis results were both good.

**Conclusion:**

The NL-SF12 is an effective measurement tool with a good reliability and acceptable validity to assess comprehensively NL for college students, and is applicable to quick, widespread use in population study and practice with low respondent burden.

## Introduction

Non-communicable diseases (NCDs) such as diabetes, cardiovascular diseases, and obesity are closely linked to unhealthy dietary behaviors (1), and these diseases are responsible for 71% of all deaths worldwide (2). Poor dietary habits are implicated in ~20% of deaths worldwide world each year (3). As a crucial influencing factor of eating behavior, nutrition literacy affected people's diet choice and health (4–7).

Nutrition literacy (NL) refers to the degree to which an individual has the capacity to obtain, process, and understand nutrition information and services required for making appropriate nutrition decisions (8–11), which is regarded as health literacy applied in the field of nutrition and an indispensable skill for the public in the twenty-first century (12). Using Nutbeam's model of health literacy (13), Velardo (8) expanded the conceptualization of nutrition and highlighted the interactive and critical NL beyond functional NL, and researchers should probably draw attention to the three dimensions. As another similar term, food literacy is often used interchangeably; in fact, food literacy is the empowerment of people to determine their food intake (14), consisting of 4 domains, i.e., planning and management; selection; preparation; and eating. Whatever, literacy skills are generally strong predictors of people's health status.

People with high NL tends to engage in healthy eating behaviors (15). For example, improved nutrition literacy can increase intake of vegetables and reduce intake of fried foods; additionally, they also encourage their family or friends with overweight or obesity, who enjoy eating high-fat or high-sugar foods, to make dietary changes (16). Recent study reported that NL along with good eating environment in college campuses improves college students' healthy eating behavior (17). Conversely, low NL was found to be associated with diet-related disorders (18, 19). Individuals with lower NL consume more high-salt, high-fat, or frying foods (16). Improving NL is regarded as a means of promoting health, particularly through enhancements in nutrition knowledge and practice (18, 20). Nutrition education has been inconsistently implemented in primary schools (21), and 66% of surveyed university students were unsure whether information on nutritional problems obtained from the internet is trusted (22, 23). Students' NL has received insufficient attention, and few effective short-form scales are available for measuring NL.

Earlier studies identified NL measurement scales with different domains (15, 24–27), or a series of specific items without considering its domains (28–30). In China, a serial of core items of NL were established for general people (31), pregnant women (32), preschool children (33), and old people (34). Based on dietary risks and dietary guidelines for Chinese people, our previous study (35) developed the NL measurement scale with 43 items (NL-43) for Chinese adults with strong reliability and validity. Previous studies stressed functional NL, nevertheless, ignored interactive and critical NL (9, 13). An individual who is of high functional NL might be able to remember or understand nutritional information, but not able to apply it if he or she is lack of interactive or critical NL. The NL-43 was characteristic of multiple features, particularly stressed interactive and critical NL, and was used as an effective tool to measure comprehensively NL for Chinese adults.

The length of questionnaire may increase participants' response burden and dimmish its acceptability for quick, widespread use in the assessment of public NL. As a result, this study was designed to develop a short-form version of the NL-43, and further assess its psychometric properties. On the basis of the study findings, the short-form scale facilitates assessments of NL levels in practice and population study.

## Study design and methods

### Study design

A cross-sectional survey was conducted in China's Anhui Province from April to May 2020. A three-stage cluster sampling strategy was used. Three cities (Bengbu, Hefei, and Wuhu located in northern, central, and southern of Anhui province, respectively) were selected by convenience sampling, and two universities (one is representative of medical university, another is representative of non-medical university) were randomly selected in each city, then two classes were randomly in each university, and in which all students were asked to take part in our survey. An individual who was 18 years old and above was included in the survey if he or she willing to participate in it, but was excluded if he or she was unwilling to do it. Considering the practical impacts of the COVID-19 pandemic on questionnaire surveying, an online questionnaire survey using Sojump, a professional online questionnaire survey platform, was conducted to collect the data. Totally, 1,359 participants finished the survey, with response rate of 96.5%.

### Questionnaire and measurement

The NL-43 was developed in accordance with the NL conceptual framework, which measures nutrition-related cognitive performance and skills, consisting of the six dimensions of knowledge, understanding, and obtaining, applying, interactive and critical skills. Knowledge refers to basic nutrition knowledge; understanding is the ability to read and comprehend nutrition information and dietary advice; obtaining skills are the ability to search for, find, and obtain nutrition information or services; applying skills are the ability to apply nutritional knowledge or nutrition services to eat a healthy diet; interactive skills are the ability to interact with food environments surrounded us under the social context and to avoid poor dietary behaviors or unhealthy food environments; and critical skills are the ability to critically reflect on nutritional information or dietary advice on the basis of individual needs. The Cronbach's α of the NL-43 is higher than 0.7. Respondents were asked to respond to a total of 43 items on a 5-point Likert scale (i.e., 1 = strongly disagree, 2 = disagree, 3 = neutral, 4 = agree, and 5 = strongly agree).

### Statistical analyses

The short-form NL scale was developed using data obtained from Chinese college students. The 1,359 samples were divided into two groups (*N*_1_ = 683 and *N*_2_ = 676) in a random method for development and validation of the short-form scale, respectively. Because validation of a short-form test should be carried out independently using independent subject samples (36, 37). Given internal consistency would be improved if each dimension had the same number of items (37), the short-form scale in this study kept two representative items in each dimension using exploratory factor analysis (EFA) and linear regression analysis.

#### Item extraction by EFA

EFA is one of the main statistical methods that can be used to shorten the number of items during the development of the short-form scale (38–40). When the Kaiser–Meyer–Olkin (KMO) measure was set at ≥0.60 and Bartlett's test of sphericity was set at a level of >0.05, the data were suitable for EFA analysis (41). Items were filtered using EFA with oblique Promax rotation. The oblique rotations accounted for the correlation between underlying variables (42). The optimal number of factors was extracted based on the eigenvalues of ≥1, and factor loadings above 0.4 were used to consider items as significantly eligible. The factor expression coefficient of each original variable was the factor loading, which reflected the effect of the extracted common factor on the original variable (41). Therefore, the short-form scale with the total of 12 items in six dimensions (Subset A) was directly developed, after selecting the first two high factor loadings in each dimension in the NL-43 scale based on the results of EFA. Meanwhile, Subset B with 24 items selected from the 6 dimensions was created based on the first four high factor loadings in each dimension. Then we created a linear regression model by using the 24 items as the independent variables and the total score of the NL-43 as the dependent variable. Two items with the first two high standardized coefficient in each dimension were selected to develop Subset C. Finally, Linear regression analysis was performed on subsets A, B, and C. The adjusted *R*^2^ values may explain the total variance in the full-form scale. In previous studies (43–45), short-form scales have been developed using linear regression analysis. *R*^2^, the coefficient of determinant in linear regression, is known as an index of the goodness of fit. The greater the goodness of fit, the more the independent variable explains the dependent variable (45).

#### Item analysis

Item analysis was conducted to ensure that the difficultly levels varied among items and that items could be distinguished among respondents. According to the total score, we divided it into high score subgroups if the score is above 73% quantiles and low score subgroup if the score is under 27% quantiles. A *t-*test was preformed to examine the differences between subgroups; if the difference was significant, then the item design for scales was appropriate; otherwise, no differentiation was evident.

#### Content validity

Content validity index (CVI), proposed and promoted by Hambleton and Martuza et al. (46, 47), was used to assess content validity. When six or more experts were involved, the item-level CVI (I-CVI) scores is no lower than 0.78, and the scale-level CVI (S-CVI; i.e., the mean of the I-CVI scores for all items), is no lower than 0.90.

#### Internal consistency

A Cronbach's α of more than 0.70 was considered to represent satisfactory reliability in assessments of internal consistency (48). Because each dimension only had two items, the Spearman–Brown coefficient was calculated to assess reliability (49).

#### Construct validity

Confirmatory factor analysis (CFA) was used to evaluate the short-form scale's construct validity and to confirm the model fit, such as root mean square of error approximation (RMSEA), with a value of <0.08 suggesting a high goodness of fit. The values of model fit indexes >0.9 were considered to represent acceptable fit. Model fit indexes included the comparative fit index (CFI), normal fit index (NFI), Tucker–Lewis index (TLI), incremental fit index (IFI), goodness-of-fit index (GFI), and adjusted goodness-of-fit index (AGFI) (50). The judgment standard refers to the standard fit of *x*^2^/d*f* < 5. Moreover, we assessed the item-scale convergent and discriminant validity based on an average variance extracted (AVE) of >0.5 and composite reliability (CR) of >0.6 (51), and we found that the square root of the AVE was greater than the correlation coefficient between dimensions, meaning that the scale had high convergent and discriminant validity (52). The short-form NL scale's criterion-related validity was evaluated using Pearson's correlation.

**Figure 1:**
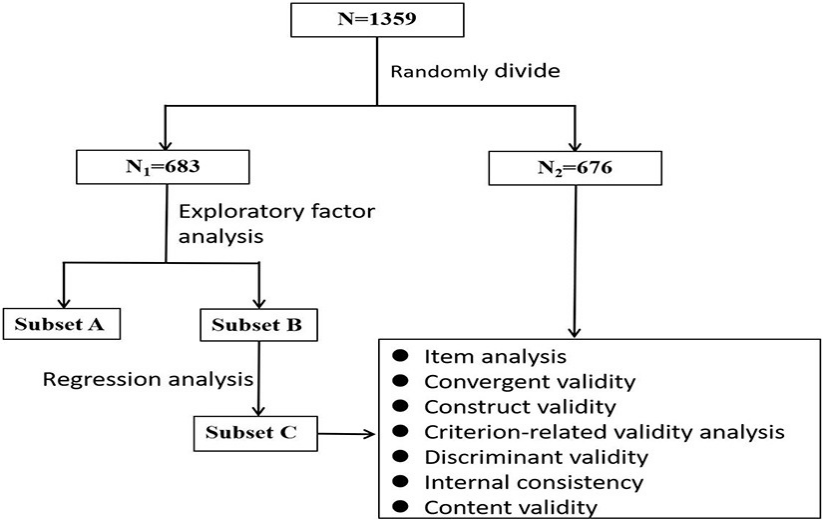
Flow chart of statistical strategies used to develop and validate the 12-item short-form nutrition literacy scale (NL-SF12).

All statistical analysis was carried out using AMOS (version 24.0) and SPSS (version 22.0). A *p-*value of <0.05 was considered statistically significant. Figure 1 illustrates the statistical strategies employed.

## Results

### Sample characteristics

As shown in Table 1, out of the 1,359 participants in this study, 553 (40.7%) and 806 (59.3%) were male and female, respectively. Of the participants, 356 (26.2%) were freshmen, 504 (37.1%) were sophomores, 230 (16.9%) were juniors, and 269 (19.8%) were seniors. furthermore, the majority of respondents were medical students (*n* = 788, 58%). The total score of the NL-SF12 was 45.34 ± 7.27 (SD), with each dimension scoring 8.44 ± 1.77 (knowledge), 7.94 ± 1.59 (understanding), 7.22 ± 1.66 (obtaining skills), 6.61 ± 1.74 (applying skills), 7.71 ± 1.45 (interactive skills), and 7.12 ± 1.56 (critical skills).

**Table 1:** Demographic characteristics of college students (*N* = 1,359).

**Characteristics**	***n***	**%**
Age/years	22.22 ± 2.94	
**Gender**		
Male	553	40.7
Female	806	59.3
**Grade**		
Freshmen	356	26.2
Sophomores	504	37.1
Juniors	230	16.9
Seniors	269	19.8
**Type of college**		
Medical	788	58
Non-medical	571	42

### Development of the short-form NL scale

EFA with oblique rotation was carried out on the first dataset (*N*1 = 683), yielding six components with eigenvalues of >1, which explained 71.4% of the variance, and the factor loadings in each component, as shown in Table 2. The *KMO* method and Bartlett's test of sphericity (*KMO* = 0.969 and χ^2^ = 27,268.968, *p* < 0.001) indicated the adequacy of EFA; in additional, positive correlations were identified between six components, ranging from 0.18 to 0.68, indicating that the Promax rotation approach was appropriate (45, 53). Afterwards, Subset A or Subset B was developed according to the first two or four high factor loadings, respectively. Then we created a linear regression model by using the 24 items in Subset B as the independent variables and the total score of the NL-43 as the dependent variable, in which Subset C was developed based on the first two high standardized coefficient in each dimension. The result from the linear regression analysis performed on Subset A and Subset C for the NL-43, respectively, indicated Subset A and Subset C had a total *R*^2^ of 0.952 and 0.964, explaining 95.2 and 96.4% of the total variance of the full-form NL-43 scale, respectively. Finally, Subset C was selected to as the short-form nutrition literacy scale termed the NL-SF12. The NL-SF12 must be evaluated for reliability and validity due to the reduction of the number of scale items and the assurance of the complete form of the simplified scale.

**Table 2:** Results of factor analysis and linear regression analysis of the 24-item subset (subset A) and the two 12-item short-form subsets (subset B and C) from the first dataset (*N*1 = 683).

**Items**	**Component**						***β***		
	**1**	**2**	**3**	**4**	**5**	**6**	**Subset A**	**Subset B**	**Subset C**
**Knowledge**									
6	0.917						0.122	0.078	0.124
2	0.914						0.132	0.066	0.119
5	0.913							0.053	
1	0.912							0.050	
4	0.898								
7	0.833								
3	0.799								
**Understanding**									
9		0.911					0.121	0.062	0.081
12		0.893					0.110	0.026	
10		0.886						0.046	
11		0.883						0.054	0.114
8		0.862							
**Obtaining skills**									
15			0.825				0.118	0.073	0.146
17			0.608				0.093	0.054	
16			0.606					0.043	
14			0.543					0.052	0.085
13			0.541						
**Applying skills**									
20				0.756			0.136	0.065	
18				0.693			0.118	0.068	
21				0.62				0.068	0.107
26				0.581				0.125	0.175
23				0.578					
19				0.575					
24				0.565					
22				0.558					
25				0.512					
27				0.527					
28				0.581					
**Interactive skills**									
30					0.893		0.107	0.060	
32					0.819		0.155	0.086	0.094
29					0.738			0.046	
33					0.733			0.065	0.088
31					0.709				
34					0.46				
37					0.44				
35					0.34				
36					0.309				
**Critical skills**									
40						0.905	0.129	0.053	
39						0.877	0.121	0.032	
38						0.872		0.060	0.124
41						0.852		0.090	0.165
42						0.827			
43						0.679			
Adjusted *R*^2^							0.952	0.982	0.964

β Standardized linear regression coefficient.

### Validation of the short-form NL scale

#### Analysis of items in the NL-SF12

Table 3 shows descriptive statistics for all items and the results based on the NL-SF12 item analysis. Significant differences were noted between high and low subgroups. This indicated that the scale item had differentiation (*T* = −37.329, *p* < 0.05) and that the value of α remained unchanged after each item was deleted.

**Table 3:** Results of the NL-SF12 item analysis and descriptive statistics for all 12 items.

**Item**	**Average**	**Standard deviation**	***t*** **-value**	**Correlation coefficient**	**Cronbach's α**
Q2	4.41	0.94	−12.025	0.549	0.889
Q6	4.33	0.94	−12.455	0.565	0.888
Q9	4.01	0.83	−23.38	0.753	0.876
Q11	3.92	0.84	−23.855	0.757	0.876
Q14	3.89	0.85	−24.07	0.779	0.874
Q15	3.32	1.05	−18.113	0.685	0.881
Q21	3.35	1.06	−14.966	0.559	0.89
Q26	3.26	1.01	−17.66	0.666	0.882
Q32	3.85	0.78	−19.215	0.712	0.879
Q33	3.86	0.82	−17.369	0.674	0.881
Q38	3.59	0.83	−22.804	0.744	0.876
Q41	3.53	0.82	−22.677	0.728	0.877

Correlation coefficient: Correlation coefficient between each item and the total score.

Cronbach's α: The value of α after the item was deleted.

Q2 : Balanced diet and reasonable nutrition are important measures to prevent and control chronic diseases such as diabetes and hypertension.

Q6 : Steaming and boiling are healthier ways of cooking than frying and grilling.

Q9 : can easily understand the nutritional information delivered by new and traditional media.

Q11 : have a good understanding of expert consensus regarding nutrition or dietary information.

Q14 : know where to find healthy diet information.

Q15 : often read nutrition information transmitted through new media (e.g., WeChat and microblogging) or watch nutrition-related programs.

Q21 : drink milk or dairy products every day.

Q26 : often buy foods based on nutrition facts on food packages.

Q32 : am open to reasonable nutrition and health advice from family or friends.

Q33 : If my family members or friends are overweight and enjoy eating high-fat foods,: will encourage them to make dietary changes.

Q38 : can easily tell whether my daily diet is reasonable.

Q41 : can estimate the suitable food intake for maintaining a healthy body weight.

#### Content validity

The CVI for each NL-SF12 module was higher than 0.9, indicating that each domain accurately reflected the meaning of NL.

#### Internal consistency

The Cronbach's α for the NL-SF12 was 0.890, and the Spearman–Brown coefficient for each dimension ranged from 0.589 to 0.890, suggesting strong internal consistency reliability.

#### Construct validity

In the second data set (*N*_2_ = 676), CFA was preformed to evaluate the structural validity of the NL-SF12. Table 4 shows good model–data fit and convergent validity. The RMSEA value of the NL-SF12 was 0.069, and the GFI, AGFI, CFI, IFI, TLI, and NFI values ranged from 0.919 to 0.972. Table 5 presents the convergent validity results. In the majority of dimensions, the AVE and CR values were above 0.5 and 0.6, respectively, with the exception of the dimension of applying skills, where the AVE value was 0.447. Table 6 shows the square root in AVE and the correlation coefficients between dimensions. In the obtaining skills (0.738) and applying skills (0.669) dimensions, discrimination validity was insufficient. The correlation between the total NL-SF12 and NL-43 scores was satisfactory, with a correlation coefficient of 0.969 (a satisfactory criterion-related validity).

**Table 4:** Construct validity for the goodness-of-fit indices of the NL-SF12.

***X*^2^/d*f***	**RMSEA**	**GFI**	**AGFI**	**CFI**	**IFI**	**TLI**	**NFI**
4.209	0.069	0.960	0.919	0.972	0.972	0.953	0.964

**Table 5:** AVE and CR values for the six dimensions of the NL-SF12.

	**AVE**	**CR (Spearman-Brown coefficient)**
Knowledge	0.789	0.881
Understanding	0.801	0.890
Obtaining skills	0.545	0.696
Applying skills	0.447	0.589
Interactive skills	0.649	0.785
Critical skills	0.770	0.870

**Table 6:** Results of the correlation analysis between the dimensions and full-form NL-SF12.

**Nutrition literacy domain**	**Knowledge**	**Understanding**	**Obtaining skills**	**Applying skills**	**Interactive skills**	**Critical skills**
Knowledge	0.789^a^					
Understanding	0.522^b^	0.801^a^				
Obtaining skills	0.462^b^	0.871^b^	0.545^a^			
Applying skills	0.222^b^	0.54^b^	0.819^b^	0.447^a^		
Interactive skills	0.417^b^	0.614^b^	0.738^b^	0.684^b^	0.649^a^	
Critical skills	0.279^b^	0.596^b^	0.766^b^	0.783^b^	0.72^b^	0.77^a^
The square root of AVE	0.888	0.895	0.738	0.669	0.806	0.877

a AVE value;

b correlation coefficient between dimensions.

## Discussion

On the basis of the NL-43, we developed and validated the NL-SF12 for university or college students. To our knowledge, it is the first study to identify a simple, effective, and comprehensive nutrition literacy measurement tool for college students in China.

In our study, the NL-SF12 maintained the initial scale's conceptual framework of six dimensions. Its' structural validation was assessed using CFA, which has been widely used to evaluate the structural validity of a scale (40, 54, 55). The CVI of >0.9 indicated that the NL content was consistent. Furthermore, adequate evidence of criterion-related validity indicated that the NL-SF12 was strongly related to the NL-43 (46). In terms of discrimination validity, knowledge, understanding, interactive skills, and critical skills dimensions had negative correlations, and the items within these domains were adequately different from each other (48). In the obtaining skills and applying skills dimensions, the correlation coefficient was greater than the square root in AVE, and discrimination validity was not obvious. Our next step will be to make the items of these two domains more specific. The findings from Cronbach's α and the Spearman–Brown coefficient suggested that the responses to the NL-SF12 items were equivalent and consistent. The internal consistency reliability showed strong robustness.

EFA is used to reduce the number of items when researchers develop a short-form scale (38–40). Previous study revealed that the value of *R*^2^ and standardized coefficient obtained through regression analysis could be helpful in developing a short-form scale (56). In our study, EFA together with regression analysis were performed to ensure the short-form scale can reflect optimally the content of the original scale. Despite the reduced number of items, the original questionnaire's factor structure efficacy of nutrition literacy was not violated, and the findings were similar to those of prior study on college students' nutrition literacy (57).

In the short-form scale, some items focused on nutrition knowledge and dietary behaviors. For example, “Balanced diet and reasonable nutrition are important measures to prevent and control chronic diseases such as diabetes and hypertension” and “If my family members or friends are overweight and enjoy eating high-fat foods, I will encourage them to make dietary changes.” These items can detect the dietary risk factors associated to chronic diseases. In addition, items retained also reflect college student' apply skills in real life, e.g., “I drink milk or dairy products every day” and “I often buy foods based on nutrition facts on food packages”. College students, on average, lack applying NL knowledge and skills to build healthy eating habits. Health and education authorities should fully cooperate to make related policies and interventions to improve their nutrition literacy.

Recent studies (31–34) identified a serial of core contents for nutrition literacy which providing evidence to develop relative measurement tools. Another study (58) also developed a nutrition literacy scale consisting of 52 items for middle-school students in Chongqing. This study provided an operationalized short-form scale to comprehensively identify NL for college students in China.

## Limitations

The external validity of the NL-SF12 was limited because it was developed and validated using a single cross-sectional data from a population of college students in Anhui. Further research is required to determine the validity of the scale in other population and regions. With regard to scale simplification, a tradeoff is inevitable with regard to the information or resources that should be omitted or saved because the reliability and validity of short-form scales are frequently compromised. Furthermore, as demonstrated in this study, future research should focus on improving convergent and discriminant validity in the dimensions of obtaining and applying skills.

## Conclusion

The NL-SF12 is an effective measurement tool with a good reliability and acceptable validity to assess comprehensively nutrition literacy for college students, and is applicable to quick, widespread use in population study and practice with low respondent burden. Nevertheless, more studies should be conducted to increase the generality of our findings.

## Data availability statement

The raw data supporting the conclusions of this article will be made available by the authors, without undue reservation.

## Ethics statement

The studies involving human participants were reviewed and approved by the Medical Ethics Committee of Bengbu Medical College, China (No. 2019002). The patients/participants provided their written informed consent to participate in this study.

## Author contributions

MZ and HL designed the study. GM and SH analyzed the data. TG and QS helped interpret the data. GM wrote the manuscript. All authors have reviewed and approved the final version of the manuscript.

## Funding

The work was supported by the Natural Science Research Project of Anhui Educational Committee (KJ2019A0302), 512 Talent training Project of Bengbu Medical College (BY51201203).

## Conflict of interest

The authors declare that the research was conducted in the absence of any commercial or financial relationships that could be construed as a potential conflict of interest.

## Publisher's note

All claims expressed in this article are solely those of the authors and do not necessarily represent those of their affiliated organizations, or those of the publisher, the editors and the reviewers. Any product that may be evaluated in this article, or claim that may be made by its manufacturer, is not guaranteed or endorsed by the publisher.
